# Sex Differences in Intracranial Aneurysms: A Matched Cohort Study

**DOI:** 10.3390/jpm14101038

**Published:** 2024-09-28

**Authors:** Vanessa M. Swiatek, Amir Amini, Michelle Marinescu, Claudia A. Dumitru, Lena Spitz, Klaus-Peter Stein, Sylvia Saalfeld, Ali Rashidi, I. Erol Sandalcioglu, Belal Neyazi

**Affiliations:** 1 Department of Neurosurgery, Otto-von-Guericke University, 39120 Magdeburg, Germany; vanessa.swiatek@med.ovgu.de (V.M.S.); amir.amini@med.ovgu.de (A.A.); michelle.marinescu@st.ovgu.de (M.M.); claudia.dumitru@med.ovgu.de (C.A.D.); klaus-peter.stein@med.ovgu.de (K.-P.S.); ali.rashidi@med.ovgu.de (A.R.); erol.sandalcioglu@med.ovgu.de (I.E.S.); 2 Department of Simulation and Graphics, Otto-von-Guericke University, 39106 Magdeburg, Germany; lena.spitz@ovgu.de; 3 Research Campus STIMULATE, 39106 Magdeburg, Germany; sylvia@isg.cs.uni-magdeburg.de; 4 Department of Medical Informatics, University Hospital Schleswig-Holstein Campus Kiel, 24105 Kiel, Germany

**Keywords:** sex medicine, intracranial aneurysms, subarachnoid hemorrhage, case-based reasoning

## Abstract

**Background:** Aneurysmal subarachnoid hemorrhage (SAH) predominantly affects women, accounting for 65% of cases. Women have a 1.3 times higher relative risk than men, with the incidence rising particularly in women aged 55–85 years. Women also have a higher prevalence of unruptured intracranial aneurysms (IAs), especially after the age of 50 years, and are at greater risk of aneurysm growth and rupture. This study aimed to isolate the influence of sex on rupture rate, bleeding severity, functional outcomes, and complications by using a matched cohort, while also examining the impact of sex on aneurysm localization and multiplicity. **Methods:** We utilized a retrospectively collected database of 300 patients with 511 IAs. Inclusion criteria included the availability of clinical data and 3D angiography for semi-automatic reconstruction of IA morphology. Female patients and their IA were matched with male patients according to clinical parameters and 21 morphological characteristics using an interactive visual exploration tool for multidimensional matching. **Results:** Contrary to previously published results, our study found no significant sex differences in rupture rates or vasospasm rates between male and female patients. The severity of SAH, functional outcomes, and complications such as hydrocephalus were also similar in women and men. However, women exhibited a higher prevalence of multiple aneurysms and distinct localization patterns. **Conclusions:** This study underscores the complex role of sex in IA development and rupture. Although sex-specific biological factors influence aneurysm characteristics, they do not necessarily translate into differences in clinical outcomes. Further research is needed to explore these factors and their impact on aneurysm development and management.

## 1. Introduction

Aneurysmal subarachnoid hemorrhage (SAH) demonstrates a significant female predominance [1], with 65% of cases occurring in women [2]. With an incidence rate of 7.9 per 100,000 person-years, women face a relative risk that is 1.3 times higher than that of men [3]. This risk increases with age, particularly in women between 55 and 85 years old [4]. On average, SAH occurs around the age of 55 years [2], with women experiencing the onset slightly later than men [5]. Despite this difference in onset age, symptom presentation and severity are generally comparable between male and female patients [6,7].

Unruptured intracranial aneurysms are more common in women, especially those over the age of 50 years, who are also at greater risk of aneurysm growth and rupture [5]. They have a higher rate of multiple aneurysms [8] and the localization of their IA differs from that in men [5]. Furthermore, the IAs of the anterior communicating artery seem to be larger in women with SAH than in men [5]. The prevalence and impact of vascular risk factors on rupture risk can vary by sex. While hypertension poses a similar risk for both sexes, alcohol abuse increases the SAH risk more significantly in men [9,10], whereas smoking raises the SAH risk more in women [9,11]. Some cardiovascular risk factors, such as a high BMI, hypercholesterolemia, and diabetes, may have protective effects, but a high BMI is notably associated with a reduced risk of SAH in women only [11]. The mechanisms behind these sex-specific effects remain unclear and not well studied [12], with only one hypothesis suggesting a link to reduced estrogen levels, particularly in female smokers [13].

In women over 55 years, the higher incidence of subarachnoid hemorrhage (SAH) and unruptured IA appears to coincide with the decline in estrogen levels that occurs during menopause [5]. Early menopause is linked to a higher risk of both conditions, indicating a potential protective role of estrogen against IA formation and rupture [14,15]. Hemodynamic stress at arterial bifurcations, which are common sites for aneurysms, may cause endothelial dysfunction, leading to vascular inflammation, oxidative stress, and weakened arterial walls [16]. This dysfunction, marked by reduced endothelial nitric oxide synthase, is an early step in aneurysm formation [16]. Estrogen can counteract this phenomenon by boosting nitric oxide synthase production, which is why high estrogen levels might be protective against IA formation and rupture [16].

The objective of this study was to examine the influence of sex using a matched cohort, controlling for cardiovascular risk factors and aneurysm morphology. By matching these parameters, the study aimed to isolate sex as the sole variable affecting the rate of rupture, bleeding severity, functional outcomes, and complications. Additionally, the study investigated the impact of sex on aneurysm localization and multiplicity.

## 2. Materials and Methods

This study has been conducted in alignment with the STROBE checklist (Supplemental material). For this analysis, we used a database that retrospectively included 300 patients with 511 IAs, all treated at the Department of Neurosurgery, Otto-von-Guericke University Hospital in Magdeburg, Germany, between 2000 and 2018. The study adhered to the following inclusion criteria:

1. Availability of clinical data, particularly the sex of the affected patient.
2. Availability of 3D angiography allowing for semi-automatic reconstruction of the morphological characteristics of the IA, either of the ruptured IA or, in cases of multiple incidental IAs, the largest IA of the patient.

**Figure 1:**
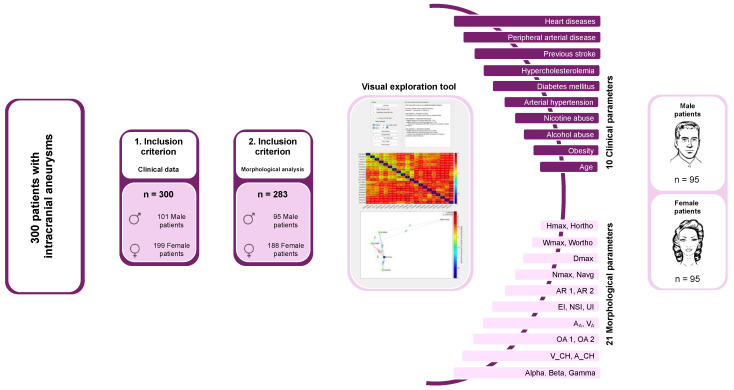
Overview of cohort formation based on inclusion criteria, along with the matching process and clinical and morphological matching criteria. The final matched cohort consisted of 95 male and 95 female patients.

Approval for the analysis of these retrospectively collected data was given by the Ethics Committee of the Otto-von-Guericke University, which noted that all procedures were part of routine care. Following the establishment of the cohort based on the inclusion criteria, female patients and their IA (defined as the ruptured IA in SAH cases; as the singular incidental IA in cases without SAH and only one IA; and as the largest incidental IA in cases without SAH and multiple IAs) were matched with each included male patient according to several clinical parameters. Additionally, 21 semi-automatically extracted morphological parameters of the IAs were included into the matching process (Figure 1).

### 2.1. Data Acquisition

For this study, clinical data were retrospectively collected by thoroughly reviewing patient’s medical records, including their medical histories, medication use, and diagnostic imaging results. The analysis included the presence of cardiovascular diseases, relevant risk factors, and other notable health conditions such as cancer or severe autoimmune disorders that needed immunosuppressive therapy. To examine the natural course and therapy of an IA, detailed information was collected from imaging studies, aneurysm-specific risk factors, and evaluations of clinical outcomes after aneurysm rupture or treatment. The parameters in this study are defined using both widely accepted standards and internal clinical definitions, originally established by Swiatek et al. [17] and adjusted to meet the specific requirements of the current study. The definitions were refined to fit the context of this research. Each parameter, along with its corresponding definition and references, are detailed below:

- Epidemiological data:
  ○ Age: defined as age at diagnosis [17].
  ○ Sex: defined as biological sex [17].
- Pre-existing conditions and risk factors:
  ○ Hypertension: documented diagnosis or intake of antihypertensive medication [17,18].
  ○ Diabetes mellitus: documented diagnosis of type 1 or 2 diabetes or intake of oral antidiabetics or insulin [17].
  ○ Hyperlipidemia: documented diagnosis or intake of medication lowering lipid or cholesterol levels in the blood [17].
  ○ Peripheral arterial disease: documented diagnosis or imaging finding [17].
  ○ Heart disease: documented diagnosis of myocardial infarction, coronary artery disease, cardiac arrhythmia, or other heart diseases [17].
  ○ Ischemic stroke: documented diagnosis or imaging finding on admission [17].
  ○ Obesity: documented body mass index of >30 kg/m^2^ [17].
  ○ Nicotine abuse: ex-nicotine abuse or continued nicotine abuse [10,17,18].
  ○ Alcohol abuse: consumption of >50 g of alcohol per week [10,17].
- Aneurysm-specific factors:
  ○ Aneurysm rupture: defined by assessment of intra-operative findings, imaging findings, and CT hemorrhage patterns indicative of aneurysm rupture [17].
  ○ Aneurysm multiplicity: defined as ≥2 intracranial aneurysms [17,19].
  ○ Aneurysm localization: defined through assessment of angiography [17].
- Clinical scores:
  ○ Hunt and Hess scale: assessment of the clinical severity of the SAH on admission based on clinical presentation, ranging from minimal symptoms to severe coma and decerebrate rigidity [20].
  ○ Fisher grade: assessment of SAH severity based on CT findings, ranging from no visible blood to significant intracerebral or intraventricular clots [21].
  ○ Modified Rankin scale at discharge: assessment of neurological and functional disability at discharge, ranging from no symptoms to death [22].
- Complications associated with aneurysm therapy/rupture:
  ○ Hydrocephalus: symptomatic enlargement of the ventricles on CT [17].
  ○ Hydrocephalus treatment: placement of an external ventricular drainage or ventriculoperitoneal shunt [17].
  ○ Vasospasm: vasospasm detection via transcranial doppler or angiography [17].
  ○ Vasospasm treatment: treatment of vasospasm via endovascular spasmolysis or other endovascular procedures [17].
  ○ Delayed cerebral ischemia: new neurological deficit or impaired consciousness lasting more than 1 h, or the appearance of new ischemia or infarcts in the affected area [17,23].
- Follow-up examinations:
  ○ Follow-up duration: measured in months starting from the first admission of the patient [17].
  ○ Occlusion of the aneurysm: assessed by follow-up imaging [17].
  ○ Modified Rankin scale at follow-up: assessment of neurological and functional disability at follow-up, ranging from no symptoms to death [22].

### 2.2. Morphological Analysis

**Figure 2:**
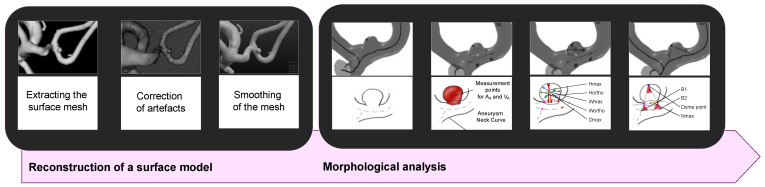
Visualization of the procedure for extracting a 3D vascular model from 3D angiography datasets, along with the subsequent morphological analysis supported by semi-automatic neck reconstruction and the determination of 21 morphological parameters [24,25].

Using the processed and digitally refined 3D rotational angiography dataset, we created 3D surface models based on the method described by Saalfeld et al. (Figure 2) [24,25]. We then applied a semi-automatic segmentation of the neck curve, which allowed us to automatically extract the following 21 morphological parameters [24,25]:

- Hmax: Maximum height of the aneurysm [25,26,27].
- Wmax: Maximum width of the aneurysm perpendicular to Hmax [25].
- Dmax: Maximum diameter of the aneurysm [25,27].
- Hortho: Height of the aneurysm; measured vertically to the aneurysm neck [25,26,27].
- Wortho: Maximum width of the aneurysm perpendicular to Hortho [25].
- Nmax: Maximum diameter of the aneurysm neck [25,26,27].
- Navg: Average diameter of the aneurysm neck [25,26,27].
- AR 1: Aspect ratio 1; (Hortho/Nmax) [25,28,29].
- AR 2: Aspect ratio 2; (Hortho/Navg) [25,28,29].
- EI: Ellipticity index; (1−18^(1/3) V_CH^(2/3)/A_CH) [26,27].
- NSI: Non-sphericity index; (1−18^(1/3) V^(2/3)/AA) [26,27].
- UI: Undulation index; (1−V/V_CH) [26,27].
- A_A_: Surface of the aneurysm [26,27].
- OA 1: Ostium Area 1; surface of the aneurysm ostium [25].
- OA 2: Ostium Area 2; surface of the aneurysm ostium; the neck curve projected onto a plane [25].
- V_A_: Volume of the aneurysm [26,27].
- V_CH: Volume of the convex hull of the aneurysm [26,27].
- A_CH: Surface of the convex hull of the aneurysm [26,27].
- Alpha: Angle at point B1 describing the angle from the baseline to the dome point [25].
- Beta: Angle at point B2 describing the angle from the baseline to the dome point [25].
- Gamma: Angle at the aneurysm dome [25].

This approach improves the objectivity of 3D vessel analysis compared to manual measurement methods and allows for the quick and efficient analysis of large data volumes.

### 2.3. Matching Criteria and Cohort Matching

To mitigate the influence of risk factors other than sex on the patients’ outcome, we conducted a systematic pairing. This approach was intended to facilitate a fair comparison of rupture rate, initial clinical presentation, aneurysm-related clinical features, and outcomes between men and women with an IA. Each man in the cohort was matched with the most similar woman and her IA. As a result, 95 men were matched with 188 women.

The matching process considered various parameters: age at diagnosis, arterial hypertension, diabetes, hypercholesterolemia, peripheral arterial disease, heart diseases, previous stroke, obesity, nicotine abuse, alcohol abuse, and 21 semi-automatically reconstructed morphological parameters (Figure 1). Given the extensive number of parameters, a tool for interactive visual exploration, developed by Spitz et al. specifically for case-based reasoning of IAs, was utilized [30].

The matching parameters were entered into the interactive visual exploration tool used in our research (Figure 1). Following each successful match, the male patient designated as the ‘patient of interest’ (POI) was sequentially replaced with the next patient, and the matching process was reinitiated with the updated set of IA patients.

We employed case-based reasoning to identify the most-similar cases of women with an IA to the selected man with an IA (the POI). The tool utilized a k-nearest-neighbor-based (k-NN) classification method to evaluate case similarity. The POI (a male patient) was matched against a reference database containing all female patients with an IA. To identify the most-similar female patient, we included the k-nearest cases, setting k to 10 in this analysis.

To ensure a fair comparison, feature values were normalized using Z-score standardization. To calculate the dissimilarity between two patients, ***x*** and ***q***, the following formula was applied:

$$dist\left(x,q\right)=\sqrt{f_{wl}\sum_{l=0}^{N}{\left(x_{l-}q_{l}\right)}^{2}}$$

where ***N*** is the number of features, ***x_l_*** represents the value of the l-th feature of patient ***x***, and ***f_wl_*** denotes the weight assigned to the feature. All feature weights were set to 1 by default.

Three variants of the k-NN-based classifier were employed in the matching process. The first classifier was a standard k-NN classifier that measured the dissimilarity between the POI and all female patients in the database, identifying the 10 nearest female patients to determine the nearest neighbors. Here, each nearest neighbor was given equal weight in the classification process, irrespective of their actual proximity to the POI. The second variant improved upon the first by weighting based on actual distances, with each nearby patient receiving a weight inversely proportional to their distance. Following a similar approach, the third variant applied min–max scaling to normalize all distances within the *[0, 1]* range.

Spitz et al. created a visual analytics framework to facilitate the interactive exploration and analysis of data. It included integrated multiple visualization tools, including a summary panel, a directed graph panel, and an interactive heat map. By using these visual tools, users were able to evaluate and compare the IA characteristics of different patients, making it easier to identify the closest match (Figure 1) [31].

**Figure 3:**
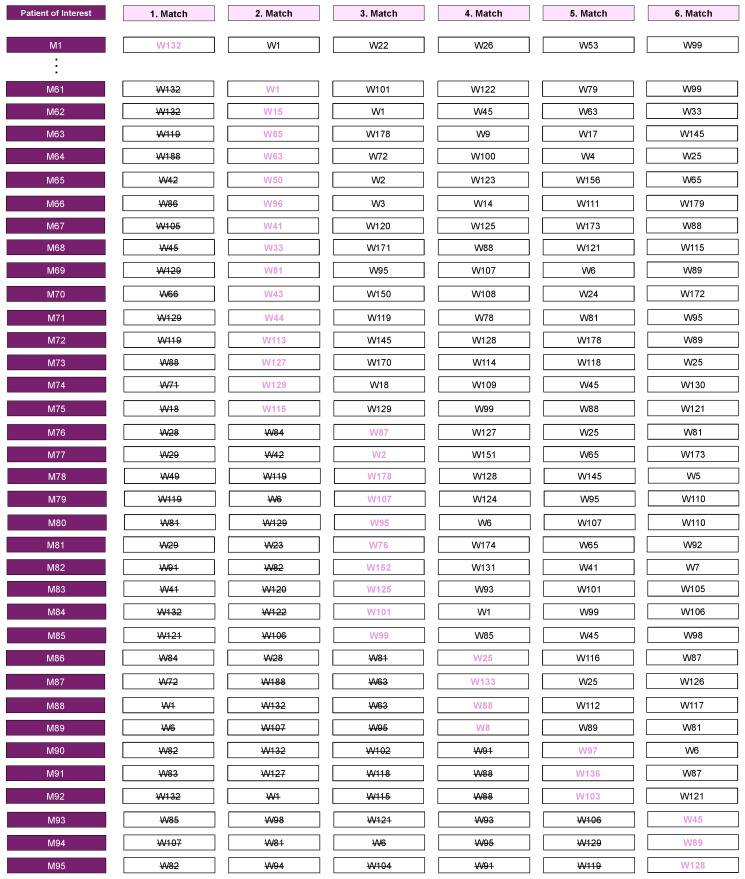
Visual representation of the matching process, focusing on the approach used when a previously matched female patient was selected as the closest match for more than one POI. In 35 instances, the initial match could not be assigned as it was already matched to another POI. To guarantee precise selection, subsequent matches were evaluated in descending order. Specifically, the second match was assigned in fifteen cases, the third match in ten cases, the fourth match in four cases, the fifth match in three cases, and the sixth match in another three cases. Pink-highlighted matches represent assigned matches, black-crossed matches denote previously matched cases, and black cases indicate non-matched cases.

For each male patient, the 10 female patients with the greatest similarity were identified during the matching process. In 35 instances, the first match could not be assigned because it was already matched to another POI and therefore included in the analysis. In such cases, the next most suitable match was selected. Specifically, the second match was assigned in fifteen cases, the third match in ten cases, the fourth match in four cases, the fifth match in three cases, and the sixth match in another three cases (Figure 3). As a result, we conducted a statistical comparison of 95 male patients with a matched cohort of 95 female patients (Figure 3).

## 3. Statistical Analysis

IBM SPSS Statistics 29 was utilized for statistical analysis. Categorial variables were analyzed using chi-square tests, with Fisher’s exact test used when any cell’s expected frequency was below five. The Kolmogorov–Smirnov test was used to test for normality in ordinal or continuous variables, followed by Levene’s test for analyzing variance homogeneity.

The Mann–Whitney U test was used for data that did not meet normal distribution criteria. For normally distributed data, either a *t*-test or Welch’s test was conducted, depending on the homogeneity of variances. The Bonferroni–Holm correction was used to correct for multiple comparisons.

## 4. Results

### 4.1. Cohort Overview

In the comprehensive cohort study involving 283 patients, a comparative analysis was performed between 95 male and 188 female patients. The findings indicated that women had a higher propensity for smoking (*p* = 0.031), whereas men exhibited a greater incidence of alcohol abuse (*p* = 0.031). There were no significant differences between the sexes concerning the prevalence of hypertension, diabetes, hypercholesterolemia, peripheral arterial disease, heart diseases, stroke, and obesity. Age distribution was also comparable between the sexes. Additionally, the 21 morphological parameters assessed showed no sex-specific distinctions (Table 1).

**Figure 4:**
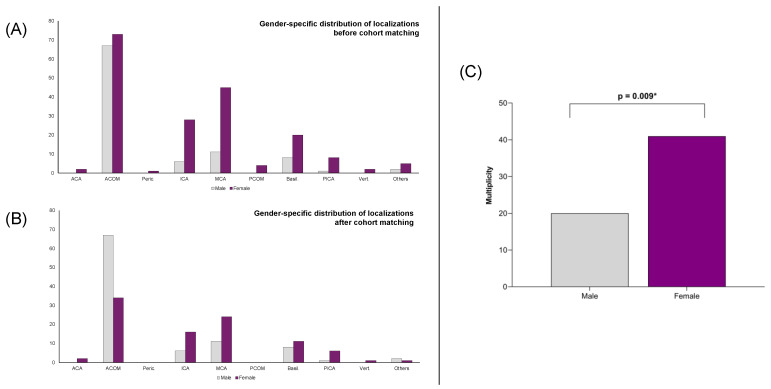
(**A**) Sex-specific distribution of IA locations before cohort matching. ACOM aneurysms were the most common in both male and female patients. However, the distribution of other locations was more heterogeneous in female patients, with many IAs located in the MCA, ICA, and basilar artery, in descending order. (**B**) Sex-specific distribution of IA locations after cohort matching. The distribution in male patients remained unchanged, with ACOM aneurysms being the most frequent. In female patients, the frequency of ACOM aneurysms decreased, and the distribution became even more heterogeneous, as previously described. (**C**) Results of the statistical analysis showing the prevalence of multiple aneurysms by sex, indicating that female patients are more frequently affected by multiple aneurysms. * highlights significant results.

Outcome analysis demonstrated no sex-related differences in aneurysm rupture rates. Among patients with ruptured aneurysms, there were no disparities in the severity of SAH between the two cohorts, as evaluated both clinically (Hunt and Hess scale) and radiologically (Fisher grade). Furthermore, functional outcomes at discharge and during follow-up, as measured by the modified Rankin scale, did not differ between male and female patients. The incidence of hydrocephalus and vasospasm was also similar across all patients. However, women presented with a higher prevalence of multiple aneurysms (*p* = 0.009), and aneurysm localization differed significantly between male and female patients (*p* = 0.009) (Figure 4). Male patients most frequently had their largest or ruptured aneurysms located at the anterior communicating artery (ACOM) (67/95; 70.5%), with other locations being uncommon. The next most frequent location was the middle cerebral artery (MCA), observed in 11 out of 95 cases (11.6%) (Figure 4). In contrast, female patients exhibited a lower proportion of ACOM aneurysms (73/188, 38.8%) and a more diverse distribution of aneurysm locations. The subsequent most common locations for female patients were the MCA (45/188, 23.9%), the internal carotid artery (ICA) (28/188, 14.9%), and the basilar artery (20/188, 10.6%) (Figure 4).

### 4.2. Analysis of the Matched Cohort

Following the matching process, the matched cohort consisting of 95 male and 95 female patients was re-evaluated to assess the consistency of the matching parameters between the two groups. The quality of the matching was confirmed, as no significant differences were observed in the matched clinical parameters (age, hypertension, diabetes, hypercholesterolemia, peripheral arterial disease, heart diseases, stroke, and obesity) or in the 21 morphological parameters between the cohorts (Table 1).

**Figure 5:**
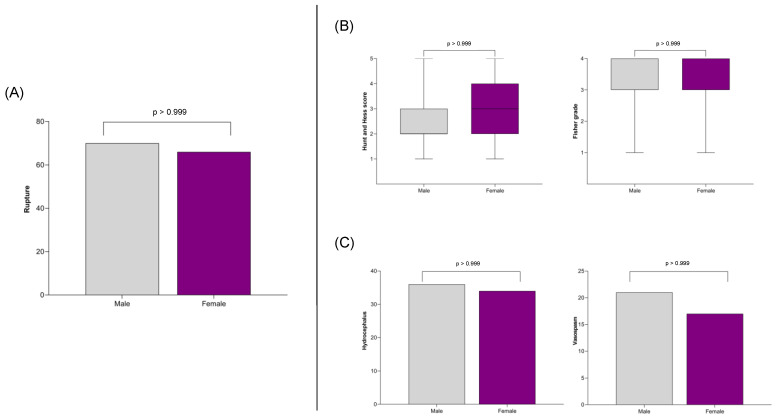
(**A**) The association between sex and rupture rate, showing no significant difference between male and female patients. (**B**) Analysis of the clinical and radiological severity of SAH also indicating no observed sex-specific difference. (**C**) Examination of the association between sex and SAH complications, specifically hydrocephalus and vasospasm, revealing no sex-specific differences.

**Figure 6:**
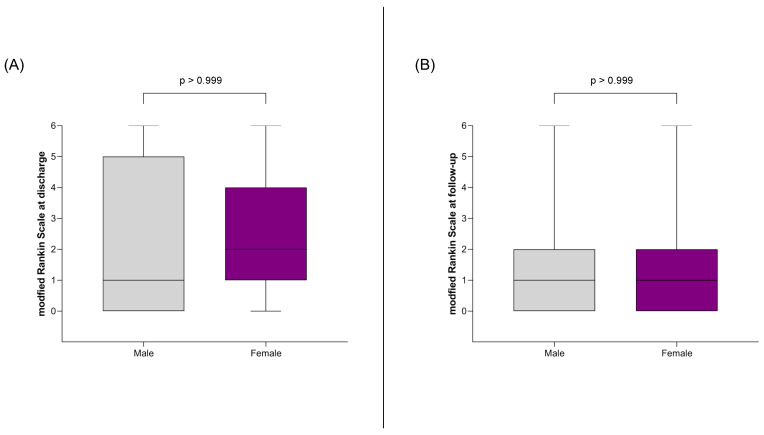
Depiction of the association between sex and clinical outcomes, measured by the mRS, at discharge (**A**) and at follow-up (**B**). No sex-specific differences were observed in either assessment.

An outcome analysis was subsequently conducted on the matched cohort, focusing on endpoints such as aneurysm rupture, clinical and radiological severity of the SAH, functional outcomes at discharge and follow-up, and the incidence of complications like hydrocephalus and vasospasm. The study also examined whether the prevalence of multiple aneurysms and the distribution of aneurysm localization differed by sex. Consistent with the findings in the non-matched cohort, there was no difference in the rupture rate between women and men (Figure 5). Male and female patients with an SAH were initially equally affected in terms of clinical severity (Hunt and Hess score) and radiological severity (Fisher grade) (Figure 5), and their functional outcomes, as measured by the mRS, were similar at discharge and follow-up (Figure 6). Complications such as hydrocephalus and vasospasm occurred with equal frequency in both cohorts (Figure 5).

The results from the non-matched cohort were further confirmed, with women continuing to show a higher prevalence of multiple aneurysms even after matching (*p* = 0.009) (Figure 4). Additionally, the distribution of aneurysm localization remained sex specific (*p* = 0.009) (Figure 4). In male patients, the largest and/or ruptured aneurysm was still most frequently located at the ACOM (67/95, 70.5%), consistent with the initial cohort. However, in female patients, the distribution of aneurysm locations remained evenly spread among common sites compared to male patients after matching. The distribution also remained stable, with aneurysms located at the ACOM (34/95, 35.8%) followed by the MCA (24/95, 25.2%), the ICA (16/95, 16.8%), and the basilar artery (11/95, 11.6%) (Figure 4).

## 5. Discussion

Understanding sex-specific differences is crucial for developing targeted prevention and treatment strategies, particularly considering the assumed protective role of estrogen against IA formation and rupture. Using a cohort of 95 male patients matched to 95 female patients based on clinical and morphological parameters, we aimed to isolate sex as the sole variable influencing the rupture rate, bleeding severity, functional outcomes, and complications. Additionally, we explored sex differences in aneurysm localization and multiplicity between male and female patients.

Aneurysmal SAH is recognized as the stroke subtype with the highest female prevalence, comprising 65% of cases [2]. Women have a higher risk of experiencing an SAH compared to men [3,32]. This increased risk is particularly noticeable in older age groups; while the incidence of SAH is similar in younger individuals, it becomes significantly higher in women aged 55–85 years, with a relative increase ranging from 1.15 to 1.5 times [3,4].

However, our findings contrast with this established understanding. We did not observe a higher rupture rate of IA in female patients in any of our analyses. One might speculate that this could be due to the inclusion of age in the matching process, potentially resulting in a cohort younger than 55 years. Nevertheless, the rupture rate did not differ between female and male patients before and after matching, despite including women aged 30–93 years in the pre-matching analysis. The mean age of the cohorts consistently centered around 55 years, the average age for SAH onset, indicating that both younger and older patients were included. Therefore, we did not observe any differences in rupture probability between the sexes before and after adjusting the cohort for age, vascular risk factors, and aneurysm morphology. Contrary to the prevailing literature, our data indicate that sex does not affect the aneurysm rupture rate.

In our study, no sex-specific differences were observed in the severity of SAHs, assessed both clinically using the Hunt and Hess score and radiologically using the Fisher grade. This aligns with previously published studies, which found no differences in the Glasgow Coma Scale on admission, or the Fisher grade, between male and female SAH patients [6].

The impact of sex on functional outcomes following an SAH is of great importance. Previous studies found no differences in mortality and functional outcomes between male and female patients [5,33,34,35,36,37,38], a finding that our analysis confirmed both before and after matching. It is important to note that the assessment of functional outcomes may not fully capture the long-term effects of an SAH, such as cognitive deficits and (neuro-)psychiatric disorders. These potential consequences were also not adequately examined in our study. Women, in particular, often experience a greater reduction in quality of life and a higher incidence of depression following an SAH [5,39]. This underscores the need for future research to focus on sex-specific differences in patients with an IA and an SAH.

When examining typical complications following an SAH, such as the occurrence of hydrocephalus and vasospasm, previous studies have yielded mixed results. While hydrocephalus rates showed no clear differences [40], earlier data indicated that women were more likely to suffer from delayed cerebral ischemia (DCI) as a result of vasospasm [5,40,41,42]. Investigations of large databases found that being female increases the risk of vasospasm, and the CONSCIOUS-1 trial also confirmed this association with higher rates of radiographic vasospasm in women [43,44]. Additionally, women aged 55–74 years are at a significantly greater risk of DCI from vasospasm compared to other age groups, a trend not seen in men [45]. In our cohort, we found no differences in the rates of hydrocephalus and vasospasm before or after matching, and consequently, no sex differences in the incidence of DCI. These findings sharply contrast with previously published data on vasospasm, underscoring the necessity for further research into the underlying mechanisms and potential sex influences on this SAH complication.

The development of multiple IAs seems to be sex specific, since women are more likely to have multiple IAs, even after adjusting for other risk factors such as smoking [8,46]. Specifically, women have a higher risk of presenting with two aneurysms (relative risk ratio 1.80) and with three or more aneurysms (relative risk ratio 3.10) [47]. These findings are consistent with those of our study, where female patients presented with more IAs even after the matching process. Furthermore, the location of the IA exhibits a distinct sex-specific pattern. Previous studies have indicated that women are more likely to have an IA along the ICA, whereas men more frequently have an IA along the anterior cerebral artery (ACA) [5,48,49]. Our study confirmed this sex-specific distribution. In male patients, the largest and/or ruptured IAs were most often located at the ACOM, consistent with prior findings. In female patients, IA locations were more evenly distributed among common sites, with the ACOM, MCA and ICA being the most frequent. These results support the established understanding of sex-specific IA localization patterns, although in our cohort, the ICA was not the most common location for an IA in women.

Sex differences in IA location and multiplicity are thought to be influenced by hormonal effects on vascular remodeling and a higher frequency of vessel wall weakness in females [5,48,49]. One study found that males have larger vessel diameters, while females experience higher wall shear stress in the MCA and ICA bifurcations, suggesting that sex-specific anatomy and hemodynamics may influence IA localization [50]. In our study, the distribution differences persisted even after matching 21 morphological parameters, indicating that factors beyond hemodynamics contribute to the sex-specific localization of IAs [51]. Further investigations are needed to fully understand these influences on aneurysm development and localization.

Our study has several limitations. First, it relies on retrospectively collected data, which may introduce selection bias. When determining the rupture status of an IA, we considered its condition at the time of presentation in our clinic and prior to any treatment. This approach means that IAs successfully treated before they could rupture were classified as unruptured in our cohort, even though they might have eventually ruptured without intervention. Our data mainly reflect the rupture rate of patients with an IA managed at our facility. However, this approach is consistent with other retrospective studies investigating the rupture risk of IAs, as prospective studies are rare due to the high morbidity and mortality associated with a SAH. Second, the matching process we employed is not a standard method. We acknowledge that due to the limited number of patients and the large number of parameters, traditional methods such as propensity score matching were not feasible. Instead, we used an innovative algorithm to match similar patients, but we recognize that this approach might not ensure the selection of “very similar” patients given the small sample size. We did not perform a formal validation, such as examining the homology of the fifth matched pair, to confirm the effectiveness of our matching algorithm, but we conducted an analysis of the matching criteria before and after matching, separated by female and male patients. Following the matching process, no discernible differences were observed between the two cohorts overall, suggesting that the cohort matching was successful and that the groups were generally comparable. Nevertheless, as mentioned earlier, this does not necessarily apply to the specific matched male and female patients. Third, while we aimed to minimize confounding variables, the matching process might not have accounted for all potential confounders that could influence functional outcomes, particularly long-term cognitive and psychiatric outcomes. This limitation is important to note because it may affect the reliability of our conclusions regarding these specific outcomes. Fourth, the study was conducted at a single center, which may limit the generalizability of our findings.

## 6. Conclusions

This study underscores the complex role of sex in the development and rupture of IAs. Contrary to previous findings, we found no significant sex differences in rupture rates, SAH severity, functional outcomes, or complications such as hydrocephalus and vasospasm after accounting for cardiovascular risk factors and aneurysm morphology between male and female patients. However, women exhibited a higher prevalence of multiple aneurysms and distinct localization patterns. These results suggest that although sex-specific biological factors influence aneurysm characteristics, they do not necessarily lead to differences in clinical outcomes. Further research, especially prospective, is essential to investigate these sex-specific factors and their impact on aneurysm development and management.

## Figures and Tables

**Table 1:** Representation of the matching criteria before and after matching, separated by female and male patients. For clinical parameters, frequencies (n and percentage) are displayed, and for morphological parameters, mean values are shown. Additionally, the table includes statistical results (*p*-values) comparing cohorts before and after matching. Post-matching, no differences were detectable, indicating successful cohort matching and establishing comparability.

	Whole Cohort (n = 283)			Matched Cohort (n = 180)		
	Male (n = 95)	Female (n = 188)	Statistical Analysis	Male (n = 95)	Female (n = 195)	Statistical Analysis
Age (mean)	55.1 years	55.6 years	*p* > 0.999 **	55.1 years	53.6 years	*p* > 0.999 ***
						
Hypertension	70 (24.7%)	134 (47.4%)	*p* > 0.999 ****	70 (73.7%)	70 (73.7%)	*p* > 0.999 ****
Diabetes mellitus	14 (5.0%)	16 (5.7%)	*p* > 0.999 ****	14 (14.7%)	10 (10.5%)	*p* > 0.999 ****
Hyperlipidemia	19 (6.7%)	29 (10.3%)	*p* > 0.999 ****	19 (20%)	18 (19.0%)	*p* > 0.999 ****
Peripheral arterial disease	4 (1.4%)	3 (1.1%)	*p* > 0.999 ****	4 (4.2%)	1 (1.1%)	*p* > 0.999 ****
Heart disease	15 (5.3%)	25 (8.8%)	*p* > 0.999 *****	15 (15.8%)	13 (13.7%)	*p* > 0.999 ****
Ischemic stroke	9 (3.2%)	11 (3.9%)	*p* > 0.999 ****	9 (9.5%)	1 (1.1%)	*p* > 0.999 ****
Obesity	33 (11.7%)	44 (15.6%)	*p* > 0.999 ****	33 (34.7%)	26 (27.4%)	*p* > 0.999 ****
Nicotine abuse	62 (21.9%)	85 (30.0%)	***p* = 0.031** ****	62 (65.3%)	68 (71.6%)	*p* > 0.999 ****
Alcohol abuse	23 (8.1%)	13 (4.6%)	***p* = 0.031** ****	23 (24.2%)	11 (11.6%)	*p* = 0.124 ****
						
Hmax (mean)	5.9	5.6	*p* > 0.999 *	5.9	5.7	*p* > 0.999 **
Wmax (mean)	5.7	5.7	*p* > 0.999 *	5.7	5.9	*p* > 0.999 **
Dmax (mean)	7.6	7.3	*p* > 0.999 *	7.6	7.4	*p* > 0.999 **
Hortho (mean)	5.0	4.9	*p* > 0.999 *	5.0	5.0	*p* > 0.999 **
Wortho (mean)	6.6	6.4	*p* > 0.999 *	6.6	6.5	*p* > 0.999 **
Nmax (mean)	4.7	4.7	*p* > 0.999 *	4.7	4.7	*p* > 0.999 **
Navg (mean)	4.1	4.0	*p* > 0.999 *	4.1	4.0	*p* > 0.999 **
AR 1 (mean)	1.1	1.1	*p* > 0.999 *	1.1	1.1	*p* > 0.999 *
AR 2 (mean)	1.2	1.3	*p* > 0.999 *	1.2	1.3	*p* > 0.999 *
EI (mean)	0.3	0.3	*p* > 0.999 **	0.3	0.3	*p* = 0.812 **
NSI (mean)	0.2	0.2	*p* > 0.999 *	0.2	0.2	*p* > 0.999 ***
UI (mean)	0.1	0.1	*p* > 0.999 *	0.1	0.1	*p* = 0.36 **
A_A_ (mean)	101.4	102.3	*p* > 0.999 *	101.4	99.3	*p* > 0.999 **
Ostium Area 1 (mean)	15.7	15.9	*p* > 0.999 *	15.6	14.6	*p* > 0.999 **
Ostium Area 2 (mean)	13.8	13.8	*p* > 0.999 *	13.8	13.1	*p* > 0.999 **
V_A_ (mean)	117.7	141.7	*p* > 0.999 *	117.7	115.8	*p* > 0.999 **
V_CH (mean)	133.2	158.6	*p* > 0.999 *	133.2	130.5	*p* > 0.999 **
A_CH (mean)	119.5	120.6	*p* > 0.999 *	119.5	117.4	*p* > 0.999 **
Alpha (mean)	76.9	76.4	*p* > 0.999 *	76.9	78.7	*p* > 0.999 *
Beta (mean)	61.4	62.6	*p* > 0.999 *	61.4	59.3	*p* > 0.999 *
Gamma (mean)	41.8	40.9	*p* > 0.999 *	41.8	42.0	*p* > 0.999 **

* *t*-test with Bonferroni–Holm correction; ** Mann–Whitney U test with Bonferroni–Holm correction; *** Welch test with Bonferroni–Holm correction; **** chi-square test with Bonferroni–Holm correction; ***** Fisher’s exact test with Bonferroni–Holm correction. Bald p-values represent significant results.

## Data Availability

The datasets obtained and analyzed during the current study are available from the corresponding author on reasonable request.

## Supplementary Materials

The following supporting information can be downloaded at: https://www.mdpi.com/article/10.3390/jpm14101038/s1, STROBE Checklist.
