# Supplementary material for: Sex Differences in Intracranial Aneurysms: A Matched Cohort Study
Source: J Pers Med. 2024 Sep 28;14(10):1038. doi: 10.3390/jpm14101038 (PMC11508385; doi:10.3390/jpm14101038)
Supplement: Supplementary file 1 [file jpm-14-01038-s001.zip › jpm-3181609-supplementary.pdf]

STROBE Statement—Checklist of items that should be included in reports of ***cohort studies***

	Item No	Recommendation	Line Number
**Title and abstract**	1	(*a*) Indicate the study's design with a commonly used term in the title or the abstract	1-2
		(*b*) Provide in the abstract an informative and balanced summary of what was done and what was found	16-35
**Introduction**			
Background/rationale	2	Explain the scientific background and rationale for the investigation being reported	38-67
Objectives	3	State specific objectives, including any prespecified hypotheses	68-72
**Methods**			
Study design	4	Present key elements of study design early in the paper	68-90
Setting	5	Describe the setting, locations, and relevant dates, including periods of recruitment, exposure, follow-up, and data collection	74-125
Participants	6	(*a*) Give the eligibility criteria, and the sources and methods of selection of participants. Describe methods of follow-up	74-125
		(*b*) For matched studies, give matching criteria and number of exposed and unexposed	74-125
Variables	7	Clearly define all outcomes, exposures, predictors, potential confounders, and effect modifiers. Give diagnostic criteria, if applicable	Tab 1
Data sources/ measurement	8*	For each variable of interest, give sources of data and details of methods of assessment (measurement). Describe comparability of assessment methods if there is more than one group	Tab 1
Bias	9	Describe any efforts to address potential sources of bias	132-182
Study size	10	Explain how the study size was arrived at	Fig 1
Quantitative variables	11	Explain how quantitative variables were handled in the analyses. If applicable, describe which groupings were chosen and why	Tab 1
Statistical methods	12	(*a*) Describe all statistical methods, including those used to control for confounding	132-199
		(*b*) Describe any methods used to examine subgroups and interactions	132-199
		(*c*) Explain how missing data were addressed	n.a.
		(*d*) If applicable, explain how loss to follow-up was addressed	n.a.
		(*e*) Describe any sensitivity analyses	n.a.
**Results**			
Participants	13*	(a) Report numbers of individuals at each stage of study—eg numbers potentially eligible, examined for eligibility, confirmed eligible, included in the study, completing follow-up, and analysed	Fig. 1
		(b) Give reasons for non-participation at each stage	Fig. 1
		(c) Consider use of a flow diagram	Fig. 1
Descriptive data	14*	(a) Give characteristics of study participants (eg demographic, clinical, social) and information on exposures and potential confounders	Tab. 2 200-208
		(b) Indicate number of participants with missing data for each variable of interest	Fig 1
		(c) Summarise follow-up time (eg, average and total amount)	n.a.
Outcome data	15*	Report numbers of outcome events or summary measures over time	200-284
Main results	16	(*a*) Give unadjusted estimates and, if applicable, confounder-adjusted estimates and their precision (eg, 95% confidence interval). Make clear	n.a.

		which confounders were adjusted for and why they were included	
		(*b*) Report category boundaries when continuous variables were categorized	n.a.
		(*c*) If relevant, consider translating estimates of relative risk into absolute risk for a meaningful time period	n.a.
Other analyses	17	Report other analyses done—eg analyses of subgroups and interactions, and sensitivity analyses	200-284
**Discussion**			
Key results	18	Summarise key results with reference to study objectives	285-360
Limitations	19	Discuss limitations of the study, taking into account sources of potential bias or imprecision. Discuss both direction and magnitude of any potential bias	361-386
Interpretation	20	Give a cautious overall interpretation of results considering objectives, limitations, multiplicity of analyses, results from similar studies, and other relevant evidence	387-397
Generalisability	21	Discuss the generalisability (external validity) of the study results	387-397
**Other information**			
Funding	22	Give the source of funding and the role of the funders for the present study and, if applicable, for the original study on which the present article is based	n.a.

*Give information separately for exposed and unexposed groups.
